# Editorial: The current challenges underlying hepatitis D virus infection

**DOI:** 10.3389/fmed.2023.1355027

**Published:** 2024-01-08

**Authors:** Romina Salpini, Valentina Svicher, Patrick T. Kennedy

**Affiliations:** ^1^Department of Biology, University of Rome Tor Vergata, Rome, Italy; ^2^Centre for Immunobiology, Blizard Institute, Barts and the London School of Medicine and Dentistry, Queen Mary, University of London, London, United Kingdom

**Keywords:** HDV, HBV, liver cirrhosis, hepatocellular carcinoma, Bulevirtide

The Hepatitis Delta virus (HDV) is the smallest human virus, causing the most severe viral hepatitis, in association with its co-helper virus, the Hepatitis B virus (HBV), on which it depends for the release of its progeny and *de-novo* entry into hepatocytes (1). Chronic HDV infection (CHD) is associated with a faster progression to cirrhosis and hepatocellular carcinoma, resulting in an increased fatality rate (2, 3). Despite its clinical relevance, CHD has been largely under-investigated for many years with relevant knowledge gaps concerning its epidemiology and pathogenic mechanisms.

This scarcity of data is explained by the limited therapeutic options available for several years. Until 2020, the only available therapeutic agent for HDV was interferon-alfa, a drug associated with a poor virological response and a high rate of post-treatment virological relapse (4). More recently, the approval of the entry inhibitor, Bulevirtide, together with the ongoing development of novel promising antiviral strategies, has led to a renewed focus around HDV and has prompted several studies aimed at better defining HDV epidemiology (5, 6). In this regard, it is relevant that the global prevalence of CHD still represents a subject of debate, with recent metanalyses reporting between 12 and 70 million HDV-infected subjects worldwide (7, 8).

Furthermore, it is noteworthy that factors underlying HDV pathogenesis are largely unknown. Particularly, the contribution of HDV-related immunological dysfunction and HDV genetic variability in modulating HDV disease progression remain poorly understood (9, 10).

Thus, this Research Topic was designed to provide new insights into HDV epidemiology and its molecular features, particularly in countries with limited data and in special high-risk populations. Moreover, the current research also examines mechanisms underlying HDV pathogenetic potential, focusing on virological and immunological factors. Overall, it comprises 7 original research articles and 3 brief research reports.

On a global perspective, there is evidence of wide heterogeneity in HDV prevalence across different areas of the world, with specific high-endemicity hospots. Among them, Mongolia is recognized as the country with the highest national anti-HDV prevalence among HBV-infected patients (ranging from 35% in the general population to 83% in high-risk groups). However, there is a paucity of studies on HDV molecular epidemiology in Mongolia. In this light, by investigating a large set of HDV sequences, Magvan et al. showed that HDV isolates from Mongolia are characterized by a remarkably high genetic variability, with multiple HDV subgenotypes-1 belonging to different clusters and still unclassified subgenotypes. Moreover, this study has revealed that one of the driving forces of HDV genetic diversity is represented by viral adptation to HLA-class-I selective pressure. This concept poses the basis for the potential selection of viral-escape variants that could challenge the success of T-cell immunotherapies under development, this will need careful consideration in future clinical trial design.

Significant data gaps on HDV seroprevalence characterize Latin America, challenging the accurate estimates of HDV circulation in this region. In central Argentina, the study by Castro et al. showed for the first time a relevant rate of HDV seropositivity (5.2%) among HBsAg carriers, with the co-circulation of HDV-genotype 1 and HBV-genotype D3, highlighting the need to promote HDV screening campaigns.

Conversely, the study led in Cuba by de los Ángeles Rodríguez Lay et al. demonstrated a completely different epidemiological scenario in this country, with a limited HDV seroprevalence (0.4%), representing a major decline respect to the 8% reported in 1988. This notable decrease can be explained by the success of the Cuban anti-HBV vaccination program introduced in 1992, confirming that, as in other countries, universal anti-HBV vaccination represents an effective strategy to constrain HBV circulation and to limit HDV spread.

Two studies, respectively from USA (Argirion et al.) and Italy (Nicolini et al.), provided new data in favor of a large HDV circulation among HBsAg+ people living with HIV (PLWH) (22% and 15% respectively), thus confirming that PLWH represents a high-risk group for HDV infection. Moreover, Nicolini et al. showed that there is a not negligible proportion of HBsAg+PLWH remaining untested for anti-HDV, thus lacking HDV diagnosis. This percentage is even higher among non-HIV HBsAg+ patients from Southern Italy (35% in Fasano et al.) reflecting suboptimal HDV diagnosis. In this light, implementing HDV screening campaigns is pivotal, especially in light of the new anti-HDV drugs on the horizon.

This issue is particularly critical considering data from the European PLWH cohorts (Swiss HIV Cohort Study, the EuroSIDA Study and the French HIV/HBV cohort), published in this Research Topic by Begrè et al., demonstrating that HDV coinfection represents the leading independent risk factor for persistent ALT elevation during long-term tenofovir treatment in PLWH. This evidence further reinforces the need to carefully monitor liver disease progression in this high-risk setting.

This Research Topic also published novel concepts on an area poorly explored: HDV-related immunopathogenesis. In particular, Joshi et al. demonstrated an increased inflammatory response characterizing patients with CHD and a weak HBV and HDV specific T-cell response, confirming the HDV role in driving a state of “immune activation,” contributing to liver damage.

Furthermore, the study from Hermanussen et al. provided new evidence on immune-mediated liver damage showing that, besides stimulating a state of immune-activation, HDV can also induce auto-immunity phenomena, that could also mediate extra-hepatic inflammatory manifestations.

Lastly, Hercun et al., by analyzing 50 liver biopsies from CHD patients, revealed for the first time the existence of a peculiar membranous HBsAg staining pattern characterizing patients on anti-HBV nucleos(t)ide analogs, that could potentially represent an indirect HBsAg diversion toward HDV replication. However, further understanding of this phenomenon, in addition to the role exerted by anti-HBV treatment in HDV co-infection is mandated to improve our understanding of HDV pathophysiology.

Overall, these articles depict a variegated and evolving epidemiological scenario for HDV infection, underlining the need to expand HDV screening programs worlwide, in order to increase diagnoses and improve the management of patients with CHD. Furthermore, the published studies on HDV pathogenesis highlight the importance of improved knowledge of the mechanisms underlying HDV-mediated immune activation since these critical insights could provide key information in the development and optimization of novel immune-based anti-HDV therapies.

## Author contributions

RS: Writing – original draft, Writing – review & editing. VS: Writing – review & editing. PK: Writing – review & editing.

## References

[1] Urban S Neumann-Haefelin C Lampertico P. Hepatitis D virus in 2021: virology, immunology and new treatment approaches for a difficult-to-treat disease. Gut. (2021) 70:1782–94. 10.1136/gutjnl-2020-323888 34103404 PMC8355886

[2] Kamal H Fornes R Simin J Stål P Duberg AS Brusselaers N. Risk of hepatocellular carcinoma in hepatitis B and D virus co-infected patients: a systematic review and meta-analysis of longitudinal studies. J Viral Hepat. (2021) 28:1431–42. 10.1111/jvh.13577 34291520

[3] Alfaiate D Clément S Gomes D Goossens N Negro F. Chronic hepatitis D and hepatocellular carcinoma: a systematic review and meta-analysis of observational studies. J Hepatol. (2020) 73:533–9. 10.1016/j.jhep.2020.02.030 32151618

[4] Sandmann L Wedemeyer H. Interferon-based treatment of chronic hepatitis D. Liver Int. (2023) 43:69–79. 10.1111/liv.15410 36002390

[5] Wedemeyer H Aleman S Brunetto MR Blank A Andreone P Bogomolov P. A phase 3, randomized trial of bulevirtide in chronic hepatitis D. N Engl J Med. (2023) 389:22–32. 10.1056/NEJMoa2213429 37345876

[6] Wedemeyer H. The burden of hepatitis D – defogging the epidemiological horizon. J Hepatol. (2020) 73:493–5. 10.1016/j.jhep.2020.06.037 32684365

[7] Stockdale AJ Kreuels B Henrion MYR Giorgi E Kyomuhangi I de Martel C. The global prevalence of hepatitis D virus infection: Systematic review and meta-analysis. J Hepatol. (2020) 73:523–32. 10.1016/j.jhep.2020.04.008 32335166 PMC7438974

[8] Chen HY Shen DT Ji DZ Han PC Zhang WM Ma JF. Prevalence and burden of hepatitis D virus infection in the global population: a systematic review and meta-analysis. Gut. (2019) 68:512–21. 10.1136/gutjnl-2018-316601 30228220

[9] Gill US. The immune landscape in hepatitis delta virus infection—Still an open field. J Viral Hepat. (2023) 30:22–6. 10.1111/jvh.13785 36529664

[10] Salpini R D'Anna S Piermatteo L Svicher V. Novel concepts on mechanisms underlying Hepatitis Delta virus persistence and related pathogenesis. J Viral Hepat. (2022) 29:1038–1047. 10.1111/jvh.13755 36256499

